# Genomic Characterization of *hox* Genes in Senegalese Sole (*Solea senegalensis*, Kaup 1858): Clues to Evolutionary Path in Pleuronectiformes

**DOI:** 10.3390/ani12243586

**Published:** 2022-12-19

**Authors:** Marco Mendizábal-Castillero, Manuel Alejandro Merlo, Ismael Cross, María Esther Rodríguez, Laureana Rebordinos

**Affiliations:** Área de Genética, Facultad de Ciencias del Mar y Ambientales, INMAR, Universidad de Cádiz, 11510 Cádiz, Spain

**Keywords:** *Solea senegalensis*, *hox* genes, cytogenomics, comparative genomics, repetitive sequences

## Abstract

**Simple Summary:**

The Senegalese sole (*Solea senegalensis*, Kaup 1858), a marine flatfish species, is of commercial interest for both fisheries and aquaculture. In aquaculture, currently there are several production bottlenecks, mainly in larvae culture and rearing. The *hox* genes participate in cell differentiation and structuring of the anterior–posterior axis during embryonic development. In this work, using cytogenetic and genomic techniques, *hox* genes were isolated and sequenced to study the sequence and cluster organization, together with the cytogenetic localization. Results were analyzed from an evolutionary perspective by comparing with other species. Our findings should represent a useful starting point for further research in order to gain more knowledge of the development of Senegalese sole for planning functional analyses focused on the main concerns that characterize the development phase.

**Abstract:**

The Senegalese sole (*Solea senegalensis*, Kaup 1858), a marine flatfish, belongs to the Pleuronectiformes order. It is a commercially important species for fisheries and aquaculture. However, in aquaculture, several production bottlenecks have still to be resolved, including skeletal deformities and high mortality during the larval and juvenile phase. The study aims to characterize the *hox* gene clusters in *S. senegalensis* to understand better the developmental and metamorphosis process in this species. Using a BAC library, the clones that contain *hox* genes were isolated, sequenced by NGS and used as BAC-FISH probes. Subsequently the *hox* clusters were studied by sequence analysis, comparative genomics, and cytogenetic and phylogenetic analysis. Cytogenetic analysis demonstrated the localization of four BAC clones on chromosome pairs 4, 12, 13, and 16 of the Senegalese sole cytogenomic map. Comparative and phylogenetic analysis showed a highly conserved organization in each cluster and different phylogenetic clustering in each *hox* cluster. Analysis of structural and repetitive sequences revealed accumulations of polymorphisms mediated by repetitive elements in the *hoxba* cluster, mainly retroelements. Therefore, a possible loss of the *hoxb7a* gene can be established in the Pleuronectiformes lineage. This work allows the organization and regulation of *hox* clusters to be understood, and is a good base for further studies of expression patterns.

## 1. Introduction

The Senegalese sole (*Solea senegalensis*, Kaup 1858) is a marine flatfish belonging to the Pleuronectiformes order and Soleidae family. It is a species of major interest in fisheries and aquaculture, mainly in the Iberian Peninsula, and is well accepted by markets throughout Europe [1]. The optimization of its culture presents several bottlenecks to be resolved, such as pathogen diseases, nutritional and digestive requirements, control of sexual differentiation and reproduction, and low quality of larvae and juveniles [2]. In recent years, genomic tools have facilitated the identification of regions associated with traits of interest for its production [3,4,5]. Particularly in the Senegalese sole, cytogenomic techniques have contributed to understanding the genome organization, leading to the description of the number and morphology of chromosomes [6,7]. Bioinformatics tools have allowed the characterization of genes and sequences of interest, such as those related to sexual differentiation, metamorphosis, immune response, histones, repetitive sequences, and transposable elements, among others [8,9,10,11,12,13,14,15]. In addition, these techniques have allowed evolutionary hypotheses to be inferred as well as the development of a high-density cytogenomic map [16,17].

In general, before they obtain their particular anatomical structure, flatfishes must undergo additional deviations from their initial body plan. Metamorphosis in flatfishes impacts directly on the bilaterality and symmetry of their external and internal structures, mainly attributed to ontogenetic changes in metamorphic remodeling [18]. It is known that fertilized eggs of Senegalese sole hatch as pelagic-living and bilaterally symmetrical larvae. However, between 9 and 19 days after hatching, metamorphosis occurs with drastic physiological and morphological changes [19]. During and after this period, a high incidence of mortality is commonly observed, together with size disparities [20]. These are caused by the appearance of skeletal and pigmentation malformations that are associated with nutritional, environmental or genetic factors [2], or the inability of homeotic mechanisms to compensate the environmental and nutritional stress during larval morphogenesis [21].

The *hox* genes are part of the *homeobox* gene family and they encode transcription factors with spatial, temporal, and hierarchical expression characteristics linked to the processes of cell differentiation and morphogenesis and closely related to the structuring of the anterior–posterior axis during embryonic development [22]. In fact, in different vertebrate groups, it has been demonstrated that paralogue *hox* genes have had a strong influence on the development of structures of the axial skeleton, such as neck, trunk, tail [23], fins, limbs [24], and spinal cord [25], all affecting the development of body plan and morphological diversity. In addition, in the teleost group, *hox* genes are expressed in the development of structures and organs [26,27,28], such as fins [29,30,31] and the pharyngeal arch [32,33,34,35].

Based on their sequence similarity, the *hox* genes derive from the duplication of an original cluster called “Proto*Hox*”, present before the divergence of Bilateral/Cnidarian symmetry organisms. However, currently, it is still not easy to describe the evolutionary pathway of *hox* genes in vertebrates. In this group, *hox* genes are divided into 4 clusters (designated a, b, c, and d), but within teleosts, these become 8 clusters (aa, ab, ba, bb, ca, cb, da, and db), which have likely arisen from a third round of genome duplication specific for the teleost lineage, called “TSGD” or 3R [36]. Based on the anterior–posterior collinearity expression of the *hox* genes, they can also be classified into four groups of paralogous genes: the anterior group (paralogues 1 and 2), group 3 (paralogue 3), the central group (paralogues 4–8) and the posterior group (paralogues 9–13) [37].

Studies of the *hox* gene in fishes are scarce and are based on the genetic and genomic characterization of the *hox* gene clusters [38,39], expression patterns [40,41,42], and one study on cytogenetics [43]. Therefore, the purpose of this work is to undertake the genomic and cytogenomic characterization of the *hox* gene clusters in *S. senegalensis* based on the structural arrangement and content of repetitive elements. The objective is to reveal molecular and evolutionary clues that would enable us to track possible differentiating structural markers in Pleuronectiformes. These, in turn, would allow further specific expression analyses of the larval development, which could be of interest for future breeding programs, to be carried out. In addition, phylogenetic and evolutionary research should enable interesting *hox* genes for such expression analysis to be found.

## 2. Materials and Methods

### 2.1. PCR Screening of hox Genes in a BAC Genomic Library of Solea senegalensis

The *hoxab*, *hoxbb*, *hoxca*, *hoxda* gene clusters were isolated from a BAC library of *Solea senegalensis* by PCR-4D methodology [44] using specific primers (Table 1). Except for the *hoxbb* cluster, the primers were designed using available sequences in the public database SoleaDB (https://www.scbi.uma.es/soleadb, accessed on 1 May 2020).

The total volume (25 µL) of PCR mix contained 50–100 ng of DNA template (BAC-DNA or genomic DNA), 4 moles µM of each primer, 4.0 mM of MgCl_2_, 1.5 U of NZYTaq II DNA polymerase enzyme, 0.4 mM of dNTP mix, plus 5 µL of 10× Reaction Buffer, and sterile H_2_O to complete. For each reaction, a negative and a positive control are included. All reagents are from Nzytech (Lisbon, Portugal). Genomic DNA was extracted from F1 specimens of the Central Service of Marine Culture Research (SC-ICM) of the University of Cádiz.

The PCR reaction was performed on the SimpliAmp™ thermal cycler from Applied Biosystems (Marsiling, Singapore). The amplification cycling consisted of an initial denaturation at 95 °C for 3 min and 35 cycles of denaturation at 95 °C for 45 s, annealing at the corresponding temperatures of each primer pair (Table 1) for 45 s, and elongation at 72 °C for 1 min. Finally, there was a last elongation step of 10 min at 72 °C. The amplified products were validated by electrophoresis in 1% agarose gel containing 0.01% ethidium bromide.

### 2.2. Extraction and Quantification of BAC Clones

Once the positive clones were detected, BAC-DNA was extracted and purified to be sequenced and used as a cytogenetic probe, using the Large Construct and Plasmid Mini kits’ protocol from Qiagen (Hilden, Germany), respectively. Once extracted, clones were quantified by spectrophotometry using the NanoDrop™ 2000/2000c (Thermo Scientific™, Waltham, MA, USA). In addition, the BAC clone was verified by PCR using the protocol previously described.

### 2.3. BAC Sequencing and Bioinformatics Analysis

BAC-DNA was sequenced by the NovaSeq 6000 method of the Illumina sequencing platform (Illumina, San Diego, CA, USA) in combination with 251 nts Paired End. Subsequently, a quality filtering was performed with the BBMap v38.36 program [46], proceeding to the elimination of those bases with a probability of error greater than 1% [47], which includes the elimination of the ends and complete sequences smaller than this value as well as those sequences smaller than 70 nucleotides in size. Sequences were continuously removed from the genomes of *Escherichia coli* str. K-12 substr. MG1655 and pCC1, using the program NGLess v1.0.0-Linux64 [48]. To perform the assembly of the sequences, the program SPAdes genome assembler v3.13.0 [49] was used, while the program QUAST v.5.0.0 [50] was used to assess the k-mer that generates the best contigs. From the contigs obtained, the semi-automated process of functional and structural annotation was carried out, in which proteins and ESTs are compared with other teleost species. All BAC clones have been deposited in the GeneBank database under the accession numbers OK504498, OK474316, OK474317, and OK504499.

### 2.4. Analysis of Repetitive Elements

An analysis of repetitive element abundance in *hox* clusters from three Pleuronectiformes species, *S. senegalensis*, *Scophthalmus maximus*, and *Cynoglossus semilaevis* as well as two other distant non-Pleuronectiformes fishes, *Sparus aurata* (Sparidae) and *Oreochromis niloticus* (Cichlidae), was carried out. The genome sequences of the five species were downloaded from the National Center for Biotechnology Information (NCBI) database: *S. senegalensis* (assembly GCA_019176455.1 IFAPA_SoseM_1); *S. maximus* (assembly ASM2237912v1); *C. semilaevis* (assembly Cse_v1.0); *S. aurata* (assembly fSpaAur1.1); and *O. niloticus* (assembly O_niloticus_UMD_NMBU). The *hox* clusters were then located and extracted from their reference genomes.

Homolog-based searches against the database CONS-Dfam_3.4 using RepeatMasker (v4.1.2) with RepBase repeat library [51] and the parameters “-s –no_is -engine rmblast –frag 20000 –species Teleostei”, were performed. The repeat elements analyzed were transposable elements (TEs) (Class I and II), simple repeats, satellite sequences, low-complexity elements and small RNA. The parameters used to measure the abundance of repeated elements of each *hox* cluster were the number of loci per Mb (NL/Mb) and their coverage, measured as a percentage of length occupied by repeated elements per cluster analyzed (%) [13,15]. To study the distribution of repetitive elements throughout the *hoxba* cluster, a sliding window of 3 kb was applied using TE annotations from RepeatMasker results. Different types of repetitive elements were recorded, such as simple repeats, DNA transposons, LINEs, SINEs, LTRs, and low-complexity sequences. Alignments of the *S. senegalensis hoxba* cluster with those of the other species were carried out with the default parameters of LASTZ software [52].

### 2.5. Comparative Genomics

Cross-species genome comparisons of the *hox* clusters were carried out using ENSEMBL and GenBank-NCBI databases in order to describe the organization within each *hox* cluster. One gene sequence from each *hox* cluster of *S. senegalensis* was extracted from the NCBI genome database and used for the BLAST searching of the *hox* cluster in another twelve representative species. These species were *Cynoglossus semilaevis* (tongue sole), *Scophthalmus maximus* (turbot), *Paralichthys olivaceus* (Japanese flounder), *Hippoglossus hippoglossus* (Atlantic halibut), *Seriola dumerili* (greater amberjack), *Seriola lalandi* (yellowtail amberjack), *Echeneis naucrates* (live sharksucker), *Oreochromis niloticus* (Nile tilapia), *Gasterosteus aculeatus* (stickleback), *Sparus aurata* (gilthead seabream), *Danio rerio* (zebrafish), and *Lepisosteus oculatus* (spotted gar). The order and direction of each gene were annotated to make a schematic representation of the cluster microsynteny.

### 2.6. BAC-FISH Mapping

Chromosome preparations were obtained from *S. senegalensis* larvae of 1–3 DPH, which were treated with 0.02% colchicine for 3 h, placed in a hypotonic solution of 0.4% KCL and fixed in modified Carnoy’s solution (absolute ethanol: acetic acid (3:1)). The cellular homogenizate was splashed onto slides previously heated to 50 °C and left until the Carnoy’s fixing was complete. Finally, the preparations were dehydrated in an increasing ethanol series of 70, 90, and 100%.

BAC probes were labeled with digoxigenin and/or biotin using DIG-Nick translation Mix or BIO-Nick translation Mix (Roche Molecular Biochemicals, Basel, Switzerland) following the manufacturer’s protocols. Hybridization and post-hybridization treatments were performed according to [53]. Finally, the plates were visualized in the Zeiss PALM MicroBeam fluorescence microscope (Jena, Germany) equipped with an AxioCam MRm digital camera (Göttingen, Germany). Imaging was by means of Axio Vision Rel. 4.8.1 software, and ZEN 3.3 blue edition software (Jena, Germany) was used for image capture and editing.

The experimental procedures were in accordance with the recommendation of the University of Cadiz (Cadiz, Spain) for the use of laboratory animals (https://bit.ly/2tPVbhY, accessed on 1 July 2022) and the Guidelines of the European Union Council (86/609/EU). The experiment was authorized by the Ethics Committee of the University of Cadiz (Cadiz, Spain).

### 2.7. Phylogenetic Analysis

A phylogenetic tree was constructed for each *hox* gene cluster. All genes were obtained from the NCBI genome database. Firstly, each paralogue of each cluster was aligned using the MAFFT program [54] following an iterative local pair method with 1000 iterations, and unmatched 5′ and 3′ ends were removed. Before paralogue concatenation, the degree of substitution saturation was tested in each gene using saturation plots with transitions (s) and transversions (v) implemented in the DAMBE6 software [55] and using GTR as the distance model. Saturation is inferred when the index of substitution saturation (ISS) is either larger than or not significantly smaller than the critical value (ISS.C). The paralogues chosen for phylogenetic analysis were those that were present in all species in addition to those not presenting a significant index of substitution saturation. The paralogues that met these requirements in each cluster were concatenated to perform the phylogenetic analysis. Twenty-three species were included to generate the phylogenetic trees (Table S1). These include five species from the Pleuronectiformes order (*S. senegalensis*, *C. semilaevis*, *S. maximus*, *H. hippoglossus*, and *P. olivaceus*), thirteen Actinopterygii teleost species, one Actinopterygii non-teleost species, one Sarcopterygii species, and three Chondrichthyes species. Sequence concatenation in each cluster was done using DAMBE6 software and aligned with the MAFFT program following an iterative “genafpair” method of 1000 iterations. The PhyML 3.0 program [56] was used to determine the best-fit phylogenetic model, and the model was then run. The statistic used for model selection was the Akaike information criterion (AIC). The resulting best-fit model in each cluster is shown in Table 2. Branch support was tested by the fast likelihood-based method using aLRT SH-like test [57]. Finally, the tree was edited in the MEGA11 program [58].

## 3. Results

### 3.1. BAC Sequencing and Annotation

Four BAC clones were isolated from the BAC library and sequenced; each contains one of the *hoxab*, *hoxbb*, *hoxca*, and *hoxda* clusters (Table 3). The total length of the sequence varied from 159.78 to 256.97 kb, and the N50 varied from 20.23 to 50.61 kb. All sequences were deposited in the NCBI database. Up to 42 genes were annotated; 21 of them were *hox* genes, and the remaining ones were genes related to other biological functions, such as protein transport, metabolism and modification, and the immune system. Three other genes have a similar function to *hox* genes, i.e., development of the anatomic structure; these are the *jazf1b*, *chn2*, and *rarga* genes.

### 3.2. Analysis of Repetitive Elements

**Figure 1:**
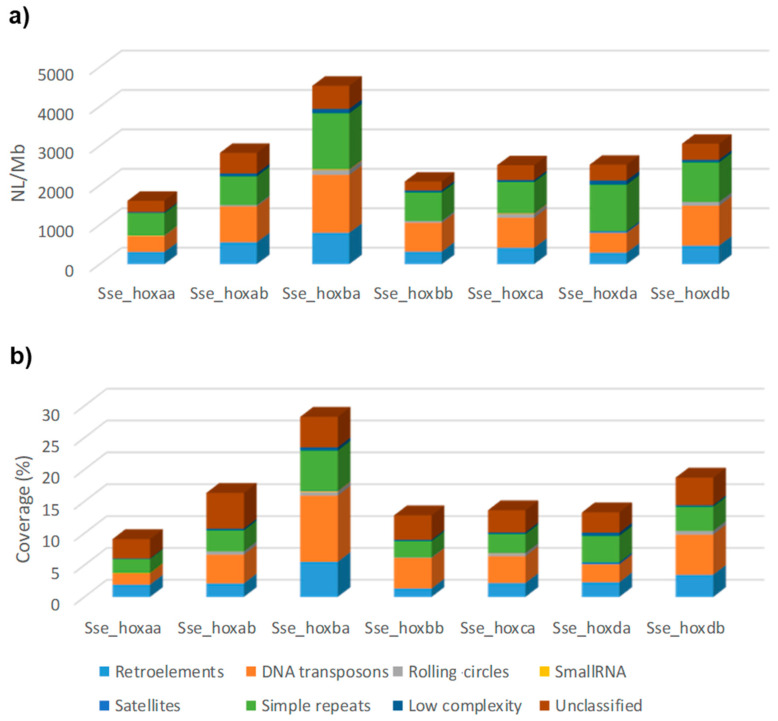
Number of *loci* per Mb (**a**) and coverage (**b**) of repetitive elements in seven *hox* clusters of the flatfish *Solea senegalensis*: Retroelements, DNA transposons, Small RNA, Rolling circles, Satellites, Simple repeats, Low complexity, and Unclassified repetitive sequences (Unknown). Coverage is measured as the percentage of length occupied by repeated elements.

**Figure 2:**
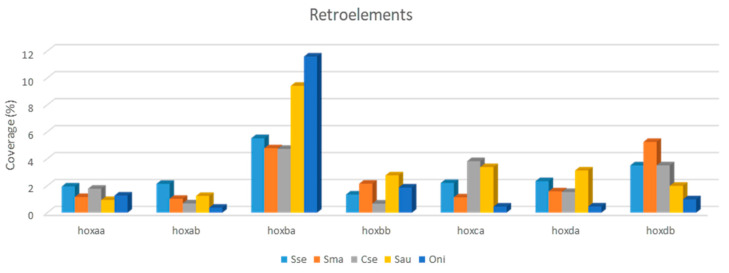
Coverage (%) of retroelements in seven *hox* clusters of five fish species: *Solea senegalensis*, *Scophthalmus maximus*, *Cynoglossus semilaevis*, *Sparus aurata*, and *Oreochromis niloticus*. Coverage is measured as the percentage of length occupied by repeated elements.

Analysis of repetitive elements in the seven *hox* clusters studied in five teleost species pointed to a larger number of *loci* and repeated DNA coverage in cluster *hoxba* (Figure 1 and Figure S1) compared with other *hox* clusters. This feature is observed mainly in the retroelement coverage (Class I transposons) (Figure 2), where the coverage values in *hoxba* were larger than in any other *hox* cluster of the five studied species (Figure S2).

**Figure 3:**
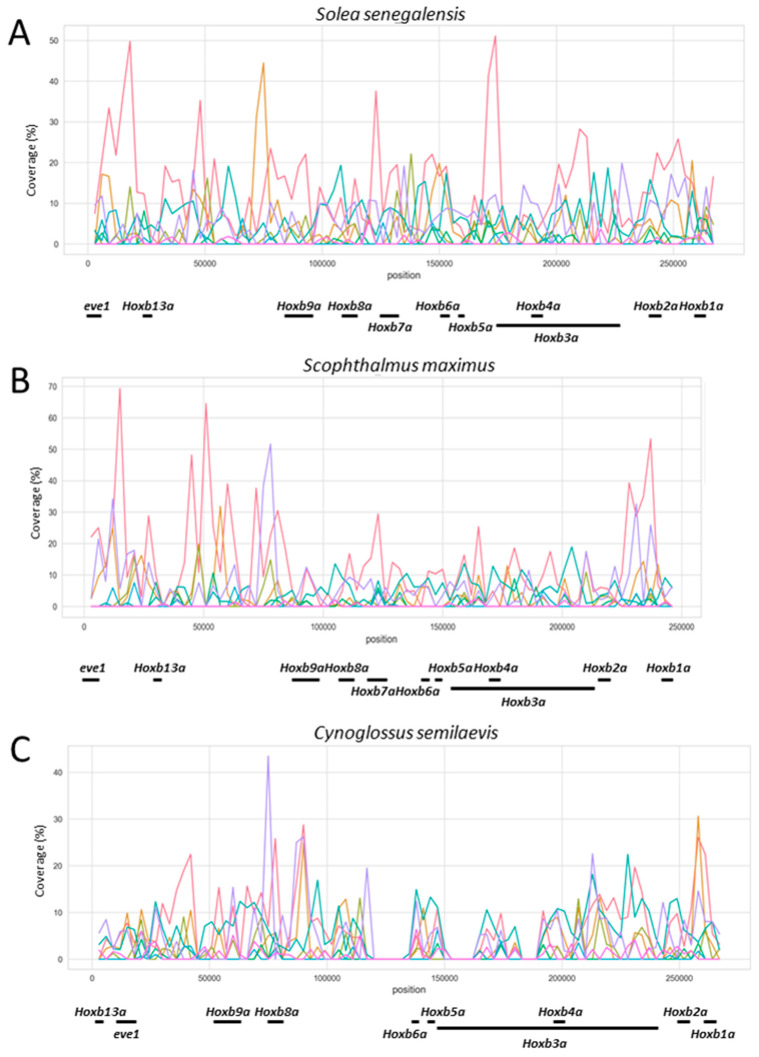
Abundance of repetitive elements measured as coverage (%) throughout the *hoxba* cluster sequence of three flatfish species (sliding window 3 kb): (**A**) *Solea senegalensis*, (**B**) *Scophthalmus maximus*, and (**C**) *Cynoglossus semilaevis*. Repetitive elements analyzed were: DNA transposons (light red), LINEs (light orange), LTRs (light green), RC/Helitrons (green), Simple repeats (blue green), SINEs (sky blue), Unknown (purple), and Low-complexity sequences (pink). Coverage is measured as the percentage of length occupied by repeated elements.

By aligning the sequence of the *S. senegalensis hoxba* cluster against sequences of the other species (Figure S3), polymorphisms can be observed in the first 120 kb (5′ region) of this *hox* cluster. In order to determine the possible relationship between this variable region and the presence of repetitive sequences, a profile of repetitive element abundance throughout the *hoxba* cluster was obtained for five fish species (Figure 3 and Figure S4). The results indicate larger coverage values in the 5′ region of the cluster in all species for several repetitive elements.

### 3.3. Cytogenetic Localization

**Figure 4:**
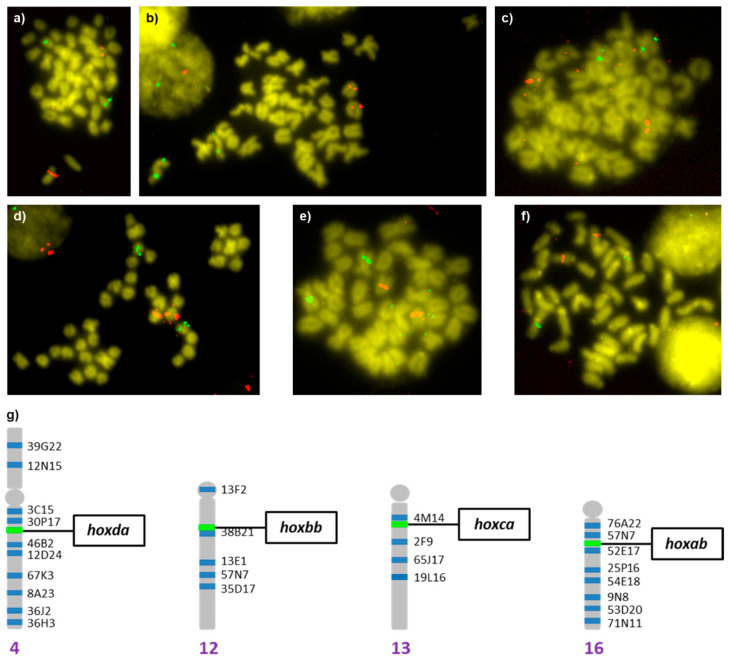
BAC-FISH among the *hox*-bearing BAC clones: (**a**) *hoxab* (red)—*hoxbb* (green); (**b**) *hoxab* (red)—*hoxca* (green); (**c**) *hoxbb* (red)—*hoxda* (green); (**d**) *hoxbb* (green)—*hoxca* (red); (**e**) *hoxab* (red)—*hoxda* (green); (**f**) *hoxca* (red)—*hoxda* (green); and (**g**) schematic representation of the localization within the cytogenetic map of *Solea senegalensis* (extracted from [17]).

The four BAC clones isolated were observed in different chromosomes pairs (Figure 4). The BAC clones 6D19, 39L15, and 51K20 were localized in acrocentric chromosome pairs, and the 29F20 was localized in a submetacentric chromosome pair. In order to ascertain the specific chromosome, each *hox*-bearing BAC clone was hybridized with chromosome markers selected from the cytogenetic map of *S. senegalensis* described by [17] (Figure S5, Figure 4g). Thus, marker BAC 54E18 allows BAC 6D19 (*hoxab* cluster) to be localized in the acrocentric pair number 16, close to the centromere. Marker BAC 38B21 helps to localize BAC 39L15 (*hoxbb* cluster) in an internal position of the acrocentric pair 12. Marker BAC 4M14 showed that the localization of BAC 51K20 (*hoxca* cluster) is in the acrocentric chromosome pair 13, close to the centromere. Finally, marker BAC 36J2 allowed BAC 29F20 (*hoxda* cluster) to be localized in a subcentromeric position of the q arm of submetacentric pair 4.

### 3.4. Comparative Genomics

**Figure 5:**
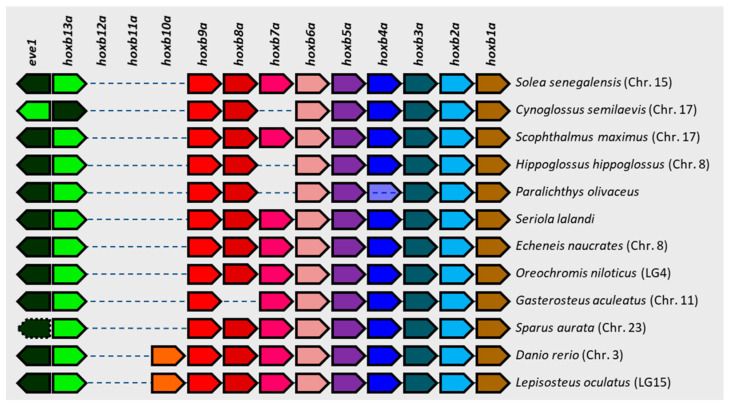
Schematic representation of the *hoxba* cluster synteny among different fish species. Each color represents the same gene in every species. Dotted box frames represent pseudogenes. Transparent boxes represent genes that were positioned by means of different scaffolds.

In addition to the localization of the above-mentioned clusters, genomic data also allowed the other three clusters to be localized. The numbering of the chromosomes follows the pattern adopted for the cytogenetic map by [17], in which we established the equivalence of the chromosome numbering between the cytogenetic map and the genome assembly IFAPA_SoseM_1. Thus, the *hoxaa* cluster is localized in chromosome 10, the *hoxba* cluster in chromosome 15, and *hoxdb* in chromosome 18. The comparative analysis of the arrangement and orientation of all *hox* genes present within each cluster showed the general rule of a conserved synteny among the different species analyzed. This was particularly evident in two *hox* clusters with a larger number of genes, i.e., in the *hoxaa* and *hoxca* clusters (Figure S6). However, the exception to this trend was the *hoxba* cluster, which presented several and varied polymorphisms, such as inversions (in *C. semilaevis*), pseudogenes (in *S. aurata*), and absences of specific genes (Figure 5). These polymorphisms were always localized in the 5′ half of the cluster, and the gene absences were mainly due to the *hoxb7a* gene in several species of Pleuronectiformes. All polymorphisms found and referred to in the general arrangement in each cluster are summarized in Table S2.

### 3.5. Phylogenetic Analysis

**Figure 6:**
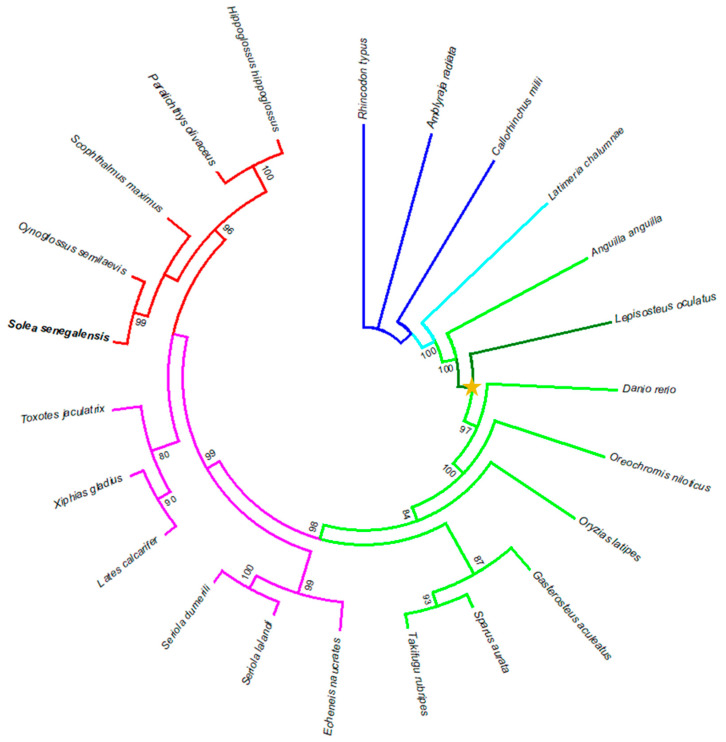
Phylogenetic tree constructed from the *hoxba* cluster (*hoxb1a*, *hoxb3a*, *hoxb6a*, *hoxb9a*, and *hoxb13a*) of twenty-two species. Blue lines: Chondrichthyes lineage; cyan lines: Sarcoptherygii lineage; dark green lines: non-Teleostei Actinopterygii lineage; pale green + pink + red lines: Teleostei lineage; pink + red lines: Carangaria lineage; and red lines: Pleuronectiformes lineage. Yellow star denotes the teleost-specific genome duplication (TSGD) event.

The phylogenetic analysis allowed the clades formed by Chrondrichthyes, Sarcoptherygii, Holostei and Teleostei to be clearly differentiated, except the cluster *hoxba*, in which one teleost species (*Anguilla anguilla*) appears before the Holostei species (*Lepisosteus oculatus*) (Figure 6). However, some other clusters could not be differentiated clearly among the different groups represented within the Teleostei clade (Figure S7).

## 4. Discussion

The recent advances achieved in the structural genomics of *S. senegalensis* [5,17,59] allow strategic research to be undertaken to resolve the most critical points for the culture of this species, i.e., those related to growth and development, including the metamorphosis process. In a previous study, 11 genes related to the metamorphosis in *S. senegalensis* were isolated from 11 BAC clones, showing a non-chromosome-specific localization [12], but *hox* genes were not included in such study. The participation of the *hox* genes in regulation of the metamorphosis has been demonstrated in other organisms, such as lampreys [60]. In this study, four new BAC chromosome markers have been located on the cytogenetic map recently published by [17], and the seven *hox* clusters present in *S. senegalensis* have been characterized. These *hox* clusters were also found in different chromosomes, thus also showing a non-chromosome-specific localization, as can be observed by FISH and genomic data in which all seven clusters are localized in different chromosome pairs. This is a general rule observed in other teleost species, as can be observed in the comparative genomic analysis. The localization of each *hox* cluster in different chromosomes is a clear evidence of the teleost-specific genome duplication (TSGD) event, which occurred approximately 350 Ma and has been cited to explain the long speciation process of teleosts [61].

Controversies have arisen concerning the association of the number of *hox* clusters with the morphological complexity and development plan along the body axis [62,63,64]. *S. senegalensis* has 49 *hox* genes, a number similar to that of other teleost species, including *C. semilaevis* (48), *E. naucartes* (49), *O. niloticus* (48), *S. aurata* (49), and *D. rerio* (48), among others, all organized in seven clusters. These numbers are similar to that of *L. oculatus* (43), a non-teleost species that did not undergo the TSGD event, so there is not a correlation with the number of paralogues and morphological complexity. Hence, the *hox* duplication from the TSDG event is not associated with an increase in the morphological complexity of the individuals but with a greater probability for the teleost species to diversify morphologically [65].

The spotted gar lineage has experienced no *hox* gene losses since its divergence from teleosts [66]. Such stability is useful for understanding the evolutionary dynamics after a duplication [67]. All *hox* genes observed in teleost species are also found in the spotted gar, but there are some other *hox* genes only present in this species (*hoxa6*, *hoxd8*, *hoxd2*, and *hoxd1*). Two of these (*hoxd8* and *hoxd1*) have been found in other super-orders of the Teleostei infraclass, so it is probable that these two genes have been lost after the radiation of the three main teleost subdivisions: Elopomorpha, Osteoglossomorpha, and Clupeocephala (this last included in this study). However, *hoxa6* and *hoxd2* could have been lost before the TSGD event or before the radiation of the extant teleost linages [68]. Comparing each of the two duplicated *hox* clusters in teleost with those corresponding homologue clusters in spotted gar, it can observed that one of the teleost duplicates conserves almost all *hox* genes that are present in the spotted gar cluster, and the other teleost duplicate has many *hox* gene losses. This observation suggests that one of the duplicated clusters has preferentially undergone gene losses that the other one has not. In contrast to other teleosts, *D. rerio* has lost the *hoxdb* cluster but has conserved the *hoxcb* cluster, which is a common characteristic among Cypriniformes [65].

The phylogenetic analysis of the *hoxba* cluster shows that an Elopomorpha teleost species such as *A. anguilla* is not clustered within the remaining Clupeocephala species: instead it appears before the holostean *L. oculatus*, thus indicating that few variations in the gene sequences of this cluster have occurred since the divergence of the evolutionary lineages of these two species. Similarly, the phylogenetic results of the *hoxbb*, *hoxca*, *hoxda*, and *hoxdb* clusters showed a different grouping of the teleost clade, also indicating the high similarity among teleost sequences in each cluster. In contrast, the *hoxaa* and *hoxab* genes accumulate enough changes to differentiate clearly each clade and subclade. This result is in accordance with that obtained by [42] based on the *hoxa2a* and *hoxa2b* phylogeny. Moreover, in a phylogenetic analysis carried out with different teleost species, *hoxba*–*hoxbb*, *hoxca*–*hoxcb* and *hoxda*–*hoxdb* clusters failed to separate a and b cluster duplicates but not with *hoxaa* and *hoxab* clusters [65], thus indicating a different divergence rate in *hoxa* paralogues except from those of the *hoxb*, *hoxc*, and *hoxd* paralogue clusters, which makes the classification based on the coding sequences of these last clusters inaccurate.

Concerning *S. senegalensis*, it presents all the *hox* genes common to all teleost species included in the study, so no *hox* losses have occurred within the Senegalese sole lineage since the divergence of the Clupeocephala super-order. However, some polymorphisms have been detected in some *hox* clusters in other species of Pleuronectiformes. These polymorphisms were observed, above all, in *P. olivaceus*, in which there was an absence of *hoxa2b*, *hoxb7a*, and *hoxc3a*. However, the polymorphisms detected in *P. olivaceus* should be treated with caution due to the poor quality in some sequence stretches. Polymorphisms have also been detected in other Pleuronectiformes species, mainly in the *hoxba* cluster, the most variable cluster. Surprisingly, all of these polymorphisms were located in the 5′ half of the cluster, and they were an inversion of *eve1* and *hoxb13a* in *C. semilaevis* and *hoxb7a* losses in *C. semilaevis*, *H. hippoglossus*, and *P. olivaceus*. Moreover, polymorphisms in this 5′ half region have also been observed in other non-Pleuronectiformes species, such the *eve1* pseudogenization in *S. aurata* and *hoxb8a* loss in *G. aculeatus*. The *hoxb7a* loss seems to be specific to some Pleuronectiformes lineages, since it has not been observed in any other species included in the study. Hence, it could be a *hox* gene that is not strictly necessary to establish the body plan of Pleuronectiformes. However, the loss of *hoxb7a* has also been described in *Tetraodon nigroviridis*, *Oryzias latipes*, *Oryzias melastigma*, *Takifugu rubripes*, *Kryptolebias marmoratus*, or *Xiphophorus maculatus* [65,68,69,70]. It has been reported that *hoxb7a* losses have occurred recurrently in particular lineages during the teleost evolution [70], the most recent event being within the radiation of East African cichlid fishes [71]. This implies that in part of the cichlid fish radiation, this gene is not essential and can be lost easily and repeatedly.

The analysis of repetitive sequences produced striking results in the *hoxba* cluster. In contrast to protostomes, repetitive elements are often completely excluded from *hox* clusters in chordates [72,73], and most vertebrate *hox* gene clusters are strongly refractory to invasion by repetitive elements [74,75,76]. However, some exceptions have been observed. Sequence comparison among the entire mouse, *Xenopus*, chicken, zebrafish, lizard, and human *hox* gene clusters revealed an abundance of repetitive elements, with a larger abundance in lizard *hox* clusters than in other species [74]. In some anole lizard species, the predominant type of interspersed repeats consists of a transposable element (TE) such as the retrotransposons [73,74]. Among vertebrates, there are qualitative and quantitative differences in TEs: Class I retrotransposons are the most abundant group in mammalian and avian genomes; and Class II DNA transposons are the most abundant transposable element in fish genomes [77]. Our results have shown that TE content in *hox* clusters is lower than whole genome coverage values [13,15,17] except in the *hoxba* cluster, where we have reported, as in the lizard species, an increased accumulation of interspersed repeats comprised mainly retrotransposons [73,74].

The size of the *hoxba* cluster observed in these fish species is also interesting because, while vertebrate *hoxb* clusters are relatively homogeneous in size (mouse, chicken, and *Xenopus* clusters are 100 kb) [73], all *hoxba* clusters analyzed in this work were found to be substantially larger, by a factor of 2.5. This larger size has been described before in lizards of genus *Anolis* due to the accumulation of repetitive elements, primarily retrotransposons, which are genetic elements typically excluded from these genomic *loci* [73,78]. In addition, teleost species such as *Gasterosteus aculeatus* and *Astatotilapia burtoni* show a large intergenic region (63 kb) containing repetitive elements between *hoxb13a* and *hoxb9a* [71]. The analysis of repetitive elements showed a higher coverage within the 5′ region of the cluster, thus being in accordance with the larger number of polymorphisms previously mentioned in this region by the comparative genomic analysis. Therefore, such polymorphisms may be motivated by the presence of these repetitive elements, primarily by retrotransposons. In addition, as noted previously, the *hoxb7a* gene has been lost independently several times during teleost evolution. The repetitive element profile analysis in Pleuronectiformes demonstrates that an accumulation of repetitive elements exists around the *hoxb7a* region, so these elements could be responsible for the losses observed for this gene in Pleuronectiformes. Overexpression of *hoxb7a* in zebrafishes has been associated with deformation of the body axis [79] and the development of a hematopoietic process in embryos [80]; this expression could be regulated by the expression of an antisense *hoxb7a* mRNA [81]. However, in zebrafishes, the *hoxb7a* gene is the only member of paralogous group 7, and so the absence of *hoxb7a* cannot be compensated for by other members of the same paralogue group [40].

TEs are known to make different functional contributions in genomes that include their roles in generating mutations, in determining genome size and rearrangements, in the regulation of gene expression, and in altering chromosome structure. In recent years, TEs have been recognized as a natural source of regulatory sequences for host genes [82,83]. Because transposable elements are a major source of genetic modifications, the accumulation of these retroelements in *hox* gene clusters may have provided an ideal substrate for the evolution of phenotypic novelties. Proliferation of TEs may have contributed to genomic incompatibilities that enabled formation of different species during evolution [78]. In addition, repetitive element densities and TE-induced changes in the regulation of *hox* genes have been correlated with speciation rates [73,78]. The role that abundance of TEs may have played in the size and polymorphisms observed in the *hoxba* clusters in the different fish species reported in this study remains unknown, and their contribution to *hox* gene cluster rearrangements in fish needs further analysis.

## 5. Conclusions

Various genetic and evolutionary characteristics of the *hox* clusters are revealed by this study. Among them is the preferential accumulation of polymorphisms in the 5′ region of the *hoxba* cluster, which is associated with a larger accumulation of repetitive elements; this could play a regulatory role in the cluster. In Pleuronectiformes, *hoxb7a* was lost in some species but not in *S. senegalensis*. All these findings have allowed us to achieve the main objective of finding differentiating structural markers in Pleuronectiformes. In turn, further directed analyses of gene expression will be focused on these structural markers, with a view to determining the role of the *hoxb7a* gene in the development of the body plan of Pleuronectiformes.

Given that studies of this kind are scarce in flatfish, the results obtained in this work represent a useful starting point for further analysis aimed at clarifying the expression pattern of these clusters in a spatial–temporal framework as well as the role played by repetitive elements in expression regulation.

## Figures and Tables

**Table 1:** Primers used to isolate the *hox* gene cluster.

Loci	Forward Primer 5′–3′	Reverse Primer 5′–3′	Ta (°C)	Reference
*hoxab*	CGGGCGAGAGAGTGGTTTATCAA	CGGAGTATCCGTGGATGAAGGAGA	64	Present work
*hoxbb*	TAYCCRAATGGSYCYGACTA	TYCKCATCCARGGRAAWATYTG	66	[45]
*hoxca*	GACCACGGGTCCCATAAGTAAT	CTCATGTCAGTGGATGAGCAGT	54	Present work
*hoxda*	CCAAACGGTCCAGAACAGCTTA	CAGTCTGCCCTTGGTGTTGG	64	Present work

**Table 2:** Selection parameters of the best-fit phylogenetic model of each *hox* gene cluster.

Cluster	Best-Fit Model	Decoration	AIC	lnL
*hoxaa*	TN93	+R	40,758.16648	−20,299.94113
*hoxab*	TN93	+R	36,039.57872	−17,927.10111
*hoxba*	GTR	+R	46,753.97854	−23,296.59684
*hoxbb*	TN93	+R	34,577.30982	−17,194.72750
*hoxca*	TN93	+R	23,974.40940	−11,912.02291
*hoxda*	GTR	+R	36,446.84214	−18,078.12721
*hoxdb*	GTR	+R	31,505.40632	−15,666.28770

**Table 3:** Sequence and annotation data from the four *hox*-bearing BAC clones.

Cluster	BAC	Total Length (bp)	N50 (bp)	L50	Annotated Genes	Accession Number
*hoxab*	6D19	256,967	30,145	4	*snx10b, skap2, **hoxa2b**, **hoxa9b**, **hoxa10b**, **hoxa11b**, **hoxa13b**, hibadhb, tax1bp1b, jazf1b, creb5b, chn2, cpvl*	OK504498
*hoxbb*	39L15	192,932	50,613	2	*skap1, **hoxb1b**, **hoxb3b**, **hoxb5b**, **hoxb6b**, ttll6, calcoco2, snf8, msl1b, chmp2a, mrm1, fbrs, srcap, stx4, arhgap44*	OK474316
*hoxca*	51K20	200,678	20,233	3	***hoxc3a*** *, **hoxc4a**, **hoxc5a**, **hoxc6a**, **hoxc8a**, **hoxc9a**, **hoxc10a**, **hoxc11a**, **hoxc12a**, **hoxc13a**, calcoco1a, rarga*	OK474317
*hoxda*	29F20	159,784	45,033	2	***hoxd4a*** *, **hoxd3a***	OK504499

## Data Availability

https://www.ncbi.nlm.nih.gov/genbank/ (accessed on 11 October 2022).

## Supplementary Materials

The following supporting information can be downloaded at: https://www.mdpi.com/article/10.3390/ani12243586/s1, Table S1: NCBI Accession Numbers of the cds used for the phylogeny; Figure S1: Number of loci per Mb and coverage of repetitive elements in seven *hox* cluster of (a) *Cynoglossus semilaevis*, (b) *Scophthalmus maximus*, (c) *Sparus aurata* and (d) *Oreochromis niloticus*. The repetitive elements analyzed were Retroelements, DNA transposons, small RNA, rolling circles, satellites, simple repeats and unclassified repetitive sequences (Unknown); Figure S2: Coverage (%) of (a) DNA transposons, (b) satellites, (c) rolling circles, (d) simple repeats, (e) small RNA, (f) low complexity and (g) unclassified repetitive sequences (Unknown) in seven *hox* cluster of five fish species: *Solea senegalensis*, *Scophthalmus maximus*, *Cynoglossus semilaevis*, *Sparus aurata* and *Oreochromis niloticus*; Figure S3: Nucleotide dot plots of the *hoxba* cluster sequence stretch between *Solea senegalensis* and (a) *Scophthalmus maximus*, (b) *Sparus aurata*, (c) *Cynoglossus semilaevis* or (d) *Oreochromis niloticus*; Figure S4: Abundance of repetitive elements, measured as coverage (%), along the *hoxba* cluster sequence of two teleost species (sliding window 3kb): (a) *Sparus aurata* and (b) *Oreochromis niloticus*. Repetitive elements analyzed were: DNA transposon (light red), LINEs (light orange), LTRs (light green), RC/Helitron (green), Simple repeat (blue-green), SINE (sky-blue), Unknown (purple) and low complexity sequences (pink); Figure S5: BAC-FISH of among *hox*-bearing BAC clones and BAC probes used as chromosome markers for *Solea senegalensis* karyotype: (a) *hoxab* (red)-BAC 54E18 (green); (b) *hoxbb* (green)-BAC 38B21 (red); (c) *hoxca* (green)-BAC 4M14 (red); (d) *hoxda* (green)-BAC 36J2 (red); Figure S6: Schematic representation of the (a) *hoxaa*, (b) *hoxab*, (c) *hoxbb*, (d) *hoxca*, (e) *hoxda* and (f) *hoxdb* clusters synteny among different fish species. Same colors represent same genes. Transparent boxes represent genes that were positioned by means of different scaffolds; Table S2: Polymorphisms detected within teleost species referred to the consensus arrangement of each cluster; Figure S7: Phylogenetic trees constructed from clusters *hoxaa* (*hoxa1a*, *hoxa3a*, *hoxa4a*, *hoxa5a*, *hoxa9a*, *hoxa11a*, *hoxa13a*, *evx1*), *hoxab* (*hoxa9b*, *hoxa10b*, *hoxa11b*, *hoxa13b*), *hoxbb* (*hoxb1b*, *hoxb5b*), *hoxca* (*hoxc5a*, *hoxc6a*, *hoxc8a*, *hoxc9a*, *hoxc10a*, *hoxc11a*, *hoxc12a*, *hoxc13a*), *hoxda* (*hoxd3a*, *hoxd4a*, *hoxd10a*, *hoxd12a*, *evx2*) and *hoxdb* (*hoxd4b*, *hoxd9b*). Blue lines: Chondrichthyes lineage; cyan lines: Sarcoptherygii lineage; dark green lines: non-Teleostei Actinoptherygii lineage; light green + pink + red lines: Teleostei lineage; pink + red lines: Carangaria lineage; red lines: Pleuronectiformes lineage. Yellow star denotes the teleost-specific genome duplication (TSGD) event.
