# Supplementary material for: Genomic Characterization of hox Genes in Senegalese Sole (Solea senegalensis, Kaup 1858): Clues to Evolutionary Path in Pleuronectiformes
Source: Animals (Basel). 2022 Dec 19;12(24):3586. doi: 10.3390/ani12243586 (PMC9774920; doi:10.3390/ani12243586)
Supplement: Supplementary file 1 [file animals-12-03586-s001.zip › animals-1997653-SI.pdf]

Table S1. NCBI Accession Numbers of the cds used for the phylogeny.

	**hoxa1a**	**hoxa3a**	**hoxa4a**	**hoxa5a**	**hoxa9a**	**hoxa11a**	**hoxa13a**	**evx**
Solea senegalensis	XM_044053073.1	XM_044053071.1	XM_044053076.1	XM_044053074.1	XM_044052424.1	XM_044052423.1	XM_044052421.1	XM_044052420.1
Hipoglossus hippoglossus	XM_034599294.1	XM_034599289.1	XM_034599297.1	XM_034599295.1	XM_034600629.1	XM_034600489.1	XM_034600488.1	XM_034600485.1
Paralichthys olivaceus	XM_020090485.1	XM_020090478.1	XM_020090490.1	XM_020090487.1	XM_020090489.1	XM_020090488.1	XM_020090486.1	XM_020090482.1
Scopthalmus maximus	XM_035621516.2	XM_035621509.2	XM_035621521.2	XM_035621518.2	XM_035621520.2	XM_035621519.2	XM_035621517.2	XM_035620695.2
Cynoglossus semilaevis	XM_008331460.3	XM_008331457.3	XM_008331455.3	XM_008331454.3	XM_008331453.3	XM_017040992.2	XM_035621517.2	XM_008331448.3
Echeneis naucrates	XM_029513552.1	XM_029513549.1	XM_029514003.1	XM_029513665.1	XM_029513872.1	XM_029513686.1	XM_029514409.1	XM_029514683.1
Seriola lalandi	XM_023431029.1	XM_023431021.1	XM_023431027.1	XM_023431026.1	XM_023431047.1	XM_023431046.1	XM_023431101.1	XM_023431102.1
Seriola dumerili	XM_022747292.1	XM_022746931.1	XM_022747273.1	XM_022746996.1	XM_022747200.1	XM_022747381.1	XM_022747507.1	XM_022747641.1
Oreochromis niloticus	XM_005453829.4	XM_025902218.1	XM_005453825.4	XM_003448866.5	XM_003448867.5	XM_003448869.5	XM_003448870.5	XM_019350306.2
Gasterosteus acuelatus	XM_040188613.1	XM_040188611.1	XM_040188616.1	XM_040188614.1	XM_040189069.1	XM_040189494.1	XM_040189495.1	XM_040187830.1
Sparus aurata	XM_030412081.1	XM_030412077.1	XM_030412083.1	XM_030412082.1	XM_030411218.1	XM_030411377.1	XM_030411402.1	XM_030411493.1
Danio Rerio	XM_017352263.2	NM_131534.1	NM_131535.1	NM_131540.1	NM_131532.2	NM_131544.1	NM_001085494.1	NM_131249.2
Lepisosteus oculatus	XM_006636214.2	XM_015357856.1	XM_006636215.2	XM_040121442.1	XM_006636060.2	XM_006636062.2	XM_006636063.2	XM_006636064.2
Xiphias gladius	XM_040121762.1	XM_040121211.1	XM_040121212.1	XM_040121442.1	XM_040121443.1	XM_040122188.1	XM_040122187.1	XM_040122175.1
Lates calcarifer	XM_018667274.2	XM_018667166.2	XM_018667365.2	XM_018667300.2	XM_018667353.2	XM_018667321.2	XM_018667311.2	XM_051066022.1
Toxotes jacultrix	XM_041052839.1	XM_041052832.1	XM_041052835.1	XM_041052834.1	XM_041053228.1	XM_041053999.1	XM_041053560.1	XM_041053805.1
Oryzias latipes	XM_004073855.3	XM_011480872.3	XM_011480868.3	AB207979.1	NM_001104666.1	XM_004073858.3	XM_004073861.4	XM_004073862.4
Takifugu rubipes	XM_011609092.2	XM_029844659.1	XM_011609093.2	XM_029845496.1	XM_011609098.2	XM_003968984.2	XM_003968986.3	XM_003968987.2
Anguila anguila	XM_035430802.1	XM_035429976.1	XM_035429982.1	XM_035429980.1	XM_035427867.1	XM_035427866.1	XM_035428605.1	XM_035428604.1
Latimeria chalumnae	XM_005993081.2	XM_014486712.1	XM_005993086.1	XM_005993085.1	XM_005993082.2	XM_005993088.1	XM_005993090.1	XM_005993091.1
Amblyraya radiata	XM_033047630.1	XM_033039426.1	XM_033047665.1	XM_033047653.1	XM_033047583.1	XM_033047552.1	XM_033047538.1	XM_033047535.1
Rhincodon typus	XM_020526887.2	XM_048611197.1	XM_020526900.2	XM_020526899.2	XM_048611312.1	XM_020526889.2	XM_020526886.2	XM_020536619.2
Callorhinchus milii	XM_007900497.2	XM_007900492.2	XM_007900493.2	XM_007900494.2	XM_007900488.2	XM_007900484.2	XM_007900483.2	XM_007900540.1

	hoxa2b	hoxa9b	hoxa10b	hoxa11b	hoxa13b
Solea senegalensis	XM_044033439.1	XM_044034539.1	XM_044033386.1	XM_044034522.1	XM_044034543.1
Hipoglossus hippoglossus	XM_034610224.1	XM_034610226.1	XM_034610221.1	XM_034610225.1	XM_034610236.1
Paralichthys olivaceus	XM_020107684.1	XM_020092794.1	XM_020092572.1	XM_020092579.1	XM_020092802.1
Scopthalmus maximus	XM_035636648.2	XM_035636686.2	XM_035636662.2	XM_035636674.2	XM_035629046.2
Cynoglossus semilaevis	XM_008323392.3	XM_008323390.2	XM_008323389.2	XM_008323388.3	XM_008323386.3
Echeneis naucrates	XM_029504329.1	XM_029504881.1	XM_029504979.1	XM_029504837.1	XM_029504659.1
Seriola lalandi	XM_023403084.1	XM_023403097.1	XM_023403096.1	XM_023403164.1	XM_023403095.1
Seriola dumerili	XM_022768488.1	XM_022768490.1	XM_022768501.1	XM_022768479.1	XM_022768482.1
Oreochromis niloticus	XM_003454387.5	XM_003454385.5	XM_003454384.5	XM_013277030.3	XM_003454381.5
Gasterosteus acuelatus	XM_040165939.1	XM_040166346.1	XM_040166343.1	XM_040166345.1	XM_040164158.1
Sparus aurata	XM_030392801.1	XM_030394498.1	XM_030392736.1	XM_030392737.1	XM_030394543.1
Danio Rerio	NM_131106.1	XM_005157968.4	XM_005157954.4	NM_131147.1	NM_131194.2
Lepisosteus oculatus	XM_006636056.2	XM_006636060.2	XM_006636061.2	XM_006636062.2	XM_006636063.2
Xiphias gladius	XM_040118965.1	XM_040118330.1	XM_040118332.1	XM_040116839.1	XM_040118290.1
Lates calcarifer	XM_018680462.2	XM_018680450.2	XM_018688598.2	XM_018688590.2	XM_018688576.2
Toxotes jacultrix	XM_041044579.1	XM_041044823.1	XM_041044841.1	XM_041044815.1	XM_041044785.1
Oryzias latipes	XM_004077830.3	NM_001104665.1	XM_004077829.3	XM_004077828.4	XM_004077827.4
Takifugu rubipes	XM_003966101.3	XM_029838911.1	XM_003966103.3	XM_003966104.2	XM_029839402.1
Anguila anguila	XM_035409817.1	XM_035400461.1	XM_035400203.1	XM_035400328.1	XM_035387260.1
Latimeria chalumnae	XM_005993080.2	XM_005993082.2	XM_005993087.2	XM_005993088.1	XM_005993090.1
Amblyraya radiata	XM_033039426.1	XM_033047583.1	XM_033047542.1	XM_033047552.1	XM_033047538.1
Rhincodon typus	XM_020526898.2	XM_048611312.1	XM_048611236.1	XM_020526889.2	XM_020526886.2
Callorhinchus milii	XM_007900496.2	XM_007900488.2	XM_007900485.2	XM_007900484.2	XM_007900483.2

	**hoxb1a**	**hoxb3a**	**hoxb5a**	**hoxb6a**	**hoxb9a**	**hoxb13a**
Solea senegalensis	XM_044051227.1	XM_044050658.1	XM_044050659.1	XM_044050661.1	XM_044051907.1	XM_044051185.1
Hipoglossus hippoglossus	XM_034594345.1	XM_034594317.1	XM_034594349.1	XM_034594353.1	XM_034594356.1	XM_034594347.1
Paralichthys olivaceus	XM_020103654.1	XM_020103679.1	XM_020103682.1	XM_020103683.1	XM_020103664.1	XM_020103651.1
Scopthalmus maximus	XM_035616535.2	XM_035616288.2	XM_035616290.2	XM_035616291.2	XM_035616466.2	XM_035615299.1
Cynoglossus semilaevis	XM_025064707.1	XM_017040668.2	XM_025064542.1	XM_008330559.3	XM_025064731.1	XM_008330561.3
Echeneis naucrates	XM_029508103.1	XM_029507856.1	XM_029507661.1	XM_029507662.1	XM_029507920.1	XM_029508846.1
Seriola lalandi	XM_023426183.1	XM_023426164.1	XM_023426170.1	XM_023426171.1	XM_023426230.1	XM_023426222.1
Seriola dumerili	XM_022753730.1	XM_022753675.1	XM_022753682.1	XM_022753683.1	XM_022753704.1	XM_022753723.1
Oreochromis niloticus	XM_019358339.2	XM_019358338.2	XM_003446381.5	XM_019358340.2	XM_003446378.5	XM_005468651.4
Gasterosteus acuelatus	XM_040191944.1	XM_040190248.1	XM_040190250.1	XM_040190252.1	XM_040191827.1	XM_040189974.1
Sparus aurata	XM_030408440.1	XM_030406674.1	XM_030406675.1	XM_030406676.1	XM_030406487.1	XM_030406722.1
Danio Rerio	NM_131115.2	NM_131117.3	NM_131101.2	NM_131119.1	NM_131121.2	NM_001044912.1
Lepisosteus oculatus	XM_006638354.2	XM_015362327.1	XM_006638313.2	XM_015362336.1	XM_006638318.2	XM_006638319.2
Xiphias gladius	XM_040116601.1	XM_040124198.1	XM_040124199.1	XM_040124200.1	XM_040122740.1	XM_040123441.1
Lates calcarifer	XM_018698423.1	XM_018698427.2	XM_018698431.2	XM_018698432.2	XM_018698466.2	XM_018698421.2
Toxotes jacultrix	XM_041063124.1	XM_041063340.1	XM_041063218.1	XM_041063219.1	XM_041063546.1	XM_041062950.1
Oryzias latipes	XM_011478648.3	XM_023957835.1	XM_004072012.4	XM_023957838.1	XM_004071881.4	XM_023957823.1
Takifugu rubipes	XM_003964295.2	XM_029836636.1	XM_003964258.3	XM_003964257.3	XM_011603555.2	XM_003964255.2
Anguila anguila	XM_035397161.1	XM_035403788.1	XM_035397164.1	XM_035397169.1	XM_035397165.1	XM_035397162.1
Latimeria chalumnae	XM_005990053.1	XM_005990050.2	XM_005990046.2	XM_005990045.2	XM_005990041.2	XM_005990054.1
Amblyraya radiata	XM_033034952.1	XM_033034945.1	XM_033034953.1	XM_033034958.1	XM_033034954.1	XM_033034951.1
Rhincodon typus	XM_020531408.2	XM_048614599.1	XM_020531412.2	XM_020531409.2	XM_020531413.2	XM_020531396.2
Callorhinchus milii	XM_007904378.2	XM_007904364.2	XM_007904370.2	XM_007904371.2	XM_007904375.2	XM_007904360.2

	hoxb1b	hoxb5b
Solea senegalensis	XM_044014574.1	XM_044014573.1
Hipoglossus hippoglossus	XM_034570616.1	XM_034570614.1
Paralichthys olivaceus	XM_020106567.1	XM_020106568.1
Scopthalmus maximus	XM_035612398.2	XM_035612401.2
Cynoglossus semilaevis	XM_008314492.3	XM_008314452.3
Echeneis naucrates	XM_029527845.1	XM_029527121.1
Seriola lalandi	XM_023406840.1	XM_023406839.1
Seriola dumerili	XM_022743186.1	XM_022742604.1
Oreochromis niloticus	XM_003448649.5	XM_003448647.5
Gasterosteus acuelatus	XM_040176913.1	XM_040176912.1
Sparus aurata	XM_030399848.1	XM_030399847.1
Danio Rerio	NM_131142.2	NM_131537.3
Lepisosteus oculatus	XM_006638354.2	XM_006638313.2
Xiphias gladius	XM_040134617.1	XM_040135901.1
Lates calcarifer	XM_018689446.2	XM_018689447.2
Toxotes jacultrix	XM_041029554.1	XM_041029556.1
Oryzias latipes	XM_004080650.4	XM_004080652.4
Takifugu rubipes	XM_003961127.3	XM_003961079.3
Anguila anguila	XM_035403800.1	XM_035403807.1
Latimeria chalumnae	XM_005990053.1	XM_005990046.2
Amblyraya radiata	XM_033034952.1	XM_033034953.1
Rhincodon typus	XM_020531408.2	XM_020531412.2
Callorhinchus milii	XM_007904378.2	XM_007904370.2

	hoxc5a	hoxc6a	hoxc8a	hoxc9a	hoxc10a	hoxc11a	hoxc12a	hoxc13a
Solea senegalensis	XM_044038514.1	XM_044038513.1	XM_044038511.1	XM_044038510.1	XM_044038503.1	XM_044037399.1	XM_044037415.1	XM_044038825.1
Hipoglossus hippoglossus	XM_034590502.1	XM_034590500.1	XM_034590703.1	XM_034590702.1	XM_034591162.1	XM_034590701.1	XM_034590360.1	XM_034590359.1
Paralichthys olivaceus	XM_020095981.1	XM_020095980.1	XM_020078106.1	XM_020095976.1	XM_020095968.1	XM_020095971.1	XM_020095975.1	XM_020095974.1
Scopthalmus maximus	XM_035643931.2	XM_035643941.2	XM_035643938.2	XM_035643937.2	XM_047330863.1	XM_035643932.2	XM_035643936.2	XM_035643934.2
Cynoglossus semilaevis	XM_008318907.3	XM_008318906.2	XM_008318904.2	XM_008318905.2	XM_008318903.3	XM_008318901.3	XM_008318900.3	XM_008318897.3
Echeneis naucrates	XM_029507229.1	XM_029507226.1	XM_029506122.1	XM_029506037.1	XM_029506665.1	XM_029506646.1	XM_029507034.1	XM_029506736.1
Seriola lalandi	XM_023425109.1	XM_023425108.1	XM_023425106.1	XM_023425104.1	XM_023425100.1	XM_023425095.1	XM_023425084.1	XM_023425254.1
Seriola dumerili	XM_022741581.1	XM_022741570.1	XM_022741538.1	XM_022741573.1	XM_022741739.1	XM_022741748.1	XM_022741805.1	XM_022741750.1
Oreochromis niloticus	XM_003448188.4	XM_005478041.3	XM_003448186.5	XM_003448185.5	XM_003448184.3	XM_003448182.5	XM_003448181.5	XM_004070462.4
Gasterosteus acuelatus	XM_040193469.1	XM_040193467.1	XM_040193465.1	XM_040193464.1	XM_040193460.1	XM_040193462.1	XM_040194476.1	XM_040194520.1
Sparus aurata	XM_030423192.1	XM_030423191.1	XM_030423189.1	XM_030423188.1	XM_030423184.1	XM_030422665.1	XM_030422667.1	XM_030422666.1
Danio Rerio	NM_131144.2	NM_131123.1	NM_001005771.1	NM_131528.3	NM_001254944.1	NM_131165.2	NM_001110759.1	NM_131543.1
Lepisosteus oculatus	XM_006629272.2	XM_006629271.2	XM_006629270.2	XM_006629269.2	XM_006629268.2	XM_006629267.2	XM_015344107.1	XM_006629265.2
Xiphias gladius	XM_040152049.1	XM_040152047.1	XM_040152045.1	XM_040152976.1	XM_040152175.1	XM_040152174.1	XM_040152177.1	XM_040152176.1
Lates calcarifer	XM_018665902.2	XM_018665900.2	XM_018665899.2	XM_018665898.2	XM_051071212.1	XM_018665893.2	XM_018665895.2	XM_018665892.2
Toxotes jacultrix	XM_041059700.1	XM_041059688.1	XM_041059666.1	XM_041059655.1	XM_041059575.1	XM_041056775.1	XM_041058361.1	XM_041057384.1
Oryzias latipes	XM_004070714.4	XM_004070467.4	NM_001163250.1	NM_001201492.1	XM_020704671.2	XM_004070464.4	XM_004070463.4	XM_004070462.4
Takifugu rubipes	XM_011621867.2	XM_003963064.3	XM_003963065.3	XM_003963066.3	XM_003963153.2	XM_003963067.3	XM_003963069.3	XM_003963070.3
Anguila anguila	XM_035382996.1	XM_035383001.1	XM_035388349.1	XM_035383655.1	XM_035383654.1	XM_035382979.1	XM_035381351.1	XM_035382953.1
Latimeria chalumnae	XM_005995613.1	XM_005995612.2	XM_005995619.2	XM_005995620.1	XM_005995621.2	XM_005995623.2	XM_005995624.2	XM_005995625.2
Callorhinchus milii	XM_007909661.2	XM_007909660.1	XM_007909657.2	XM_007909658.1	XM_007909656.2	XM_007909653.2	XM_007909654.2	XM_007909652.2

	**hoxd3a**	**hoxd4a**	**hoxd10a**	**hoxd11a**	**hoxd12a**	**evx**
Solea senegalensis	XM_044018128.1	XM_044018137.1	XM_044051409.1	XM_044050496.1	XM_044019062.1	XM_044019140.1
Hipoglossus hippoglossus	XM_034574562.1	XM_034574563.1	XM_034574122.1	XM_034574156.1	XM_034574221.1	XM_034575001.1
Paralichthys olivaceus	XM_020089916.1	XM_020089920.1	XM_020089917.1	XM_020089921.1	XM_020089806.1	XM_020089889.1
Scopthalmus maximus	XM_035603933.2	XM_035603939.2	XM_035603934.2	XM_035603935.2	XM_035603938.2	XM_035603932.2
Cynoglossus semilaevis	XM_017039818.2	XM_008328316.3	XM_017039824.2	XM_008328310.2	XM_017039822.2	XM_008328309.3
Echeneis naucrates	XM_029529979.1	XM_029529980.1	XM_029530595.1	XM_029530622.1	XM_029530946.1	XM_029492877.1
Seriola lalandi	XM_023405695.1	XM_023405697.1	XM_023405688.1	XM_023405689.1	XM_023405693.1	XM_023405694.1
Seriola dumerili	XM_022740452.1	XM_022740454.1	XM_022740964.1	XM_022740952.1	XM_022741150.1	XM_022741205.1
Oreochromis niloticus	XM_025904031.1	XM_005452901.4	XM_003447300.5	XM_005452905.3	XM_003447301.5	XM_003447302.5
Gasterosteus acuelatus	XM_040202138.1	XM_040202139.1	XM_040202511.1	XM_040202512.1	XM_040200614.1	XM_040202546.1
Sparus aurata	XM_030429544.1	XM_030429545.1	XM_030428626.1	XM_030428631.1	XM_030428630.1	XM_030428624.1
Danio Rerio	XM_021478602.1	NM_001126445.2	NM_131166.2	NM_131167.1	NM_001126486.1	NM_131232.2
Lepisosteus oculatus	XM_006636490.2	XM_006636491.2	XM_006636493.2	XM_006636494.2	XM_006636495.2	XM_006636497.2
Xiphias gladius	XM_040148524.1	XM_040148528.1	XM_040148963.1	XM_040149453.1	XM_040149452.1	XM_040149449.1
Lates calcarifer	XM_018675791.2	XM_018675792.2	XM_018675786.2	XM_018675790.2	XM_018675788.2	XM_018675785.2
Toxotes jacultrix	XM_041057208.1	XM_041057210.1	XM_041057454.1	XM_041057425.1	XM_041057534.1	XM_041057692.1
Oryzias latipes	XM_011489801.3	AB232923.1	XM_004081767.4	XM_011489797.3	XM_004081928.4	XM_023951144.1
Takifugu rubipes	XM_029846105.1	XM_011609482.2	XM_003961830.2	XM_011609522.2	XM_003961831.2	XM_003961832.2
Anguilla anguilla	XM_035410365.1	XM_035410369.1	XM_035411116.1	XM_035411178.1	XM_035411313.1	XM_035411038.1
Latimeria chalumnae	XM_014491156.1	XM_006000588.1	XM_006000596.2	XM_006000595.2	XM_006000597.2	XM_006000598.1
Amblyraya radiata	XM_033023884.1	XM_033023894.1	XM_033023886.1	XM_033023889.1	XM_033023890.1	XM_033023899.1
Rhincodon typus	XM_048598653.1	XM_048598655.1	XM_020520271.2	XM_048598765.1	XM_020519727.2	XM_020519402.2
Callorhinchus milii	XM_007890055.2	XM_007890050.2	XM_007890043.2	XM_007890044.2	XM_007890045.2	XM_007890041.2

	hoxd0b4	hoxd9b
Solea senegalensis	XM_044045041.1	XM_044045040.1
Hipoglossus hippoglossus	XM_034609295.1	XM_034609296.1
Paralichthys olivaceus	XM_020093164.1	XM_020093177.1
Scopthalmus maximus	XM_035606243.2	XM_035606240.2
Cynoglossus semilaevis	XM_025061184.1	XM_008322471.2
Echeneis naucrates	XM_029521336.1	XM_029521333.1
Seriola lalandi	XM_023393510.1	XM_023393508.1
Seriola dumerili	XM_022752839.1	XM_022752836.1
Oreochromis niloticus	XM_003438338.5	XM_003438242.5
Gasterosteus acuelatus	XM_040178745.1	XM_040178742.1
Sparus aurata	XM_030442627.1	XM_030441114.1
Lepisosteus oculatus	XM_006636491.2	XM_006636577.2
Xiphias gladius	XM_040139702.1	XM_040139778.1
Lates calcarifer	XM_018693801.2	XM_018693803.2
Toxotes jacultrix	XM_041049176.1	XM_041049172.1
Oryzias latipes	AB208019.1	AB208020.1
Takifugu rubipes	XM_003963848.3	XM_003963805.3
Anguila anguila	XM_035392667.1	XM_035392665.1
Latimeria chalumnae	XM_006000588.1	XM_006000594.2
Amblyraya radiata	XM_033047665.1	XM_033023891.1
Rhincodon typus	XM_048598655.1	XM_048611304.1
Callorhinchus milii	XM_007890050.2	XM_007890047.2

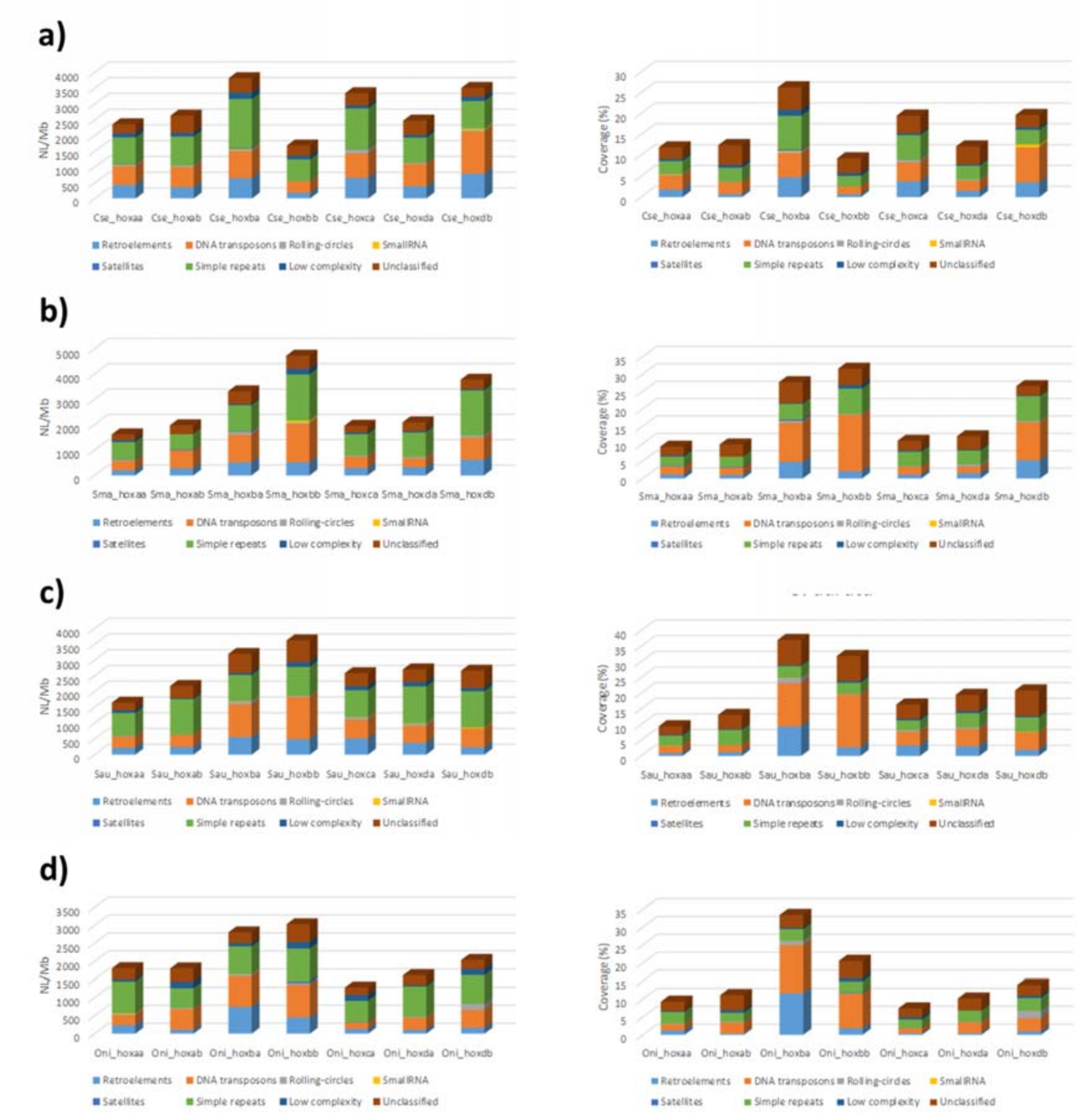

a)
NL/Mb
Coverage (%)
Cse_hoxaa Cse_hoxab Cse_hoxba Cse_hoxbb Cse_hoxca Cse_hoxda Cse_hoxdb
Retroelements
DNA transposons
Rolling-circles
SmallRNA
Satellites
Simple repeats
Low complexity
Unclassified
b)
NL/Mb
Coverage (%)
Sma_hoxaa Sma_hoxab Sma_hoxba Sma_hoxbb Sma_hoxca Sma_hoxda Sma_hoxdb
c)
NL/Mb
Coverage (%)
Sau_hoxaa Sau_hoxab Sau_hoxba Sau_hoxbb Sau_hoxca Sau_hoxda Sau_hoxdb
d)
NL/Mb
Coverage (%)
Oni_hoxaa Oni_hoxab Oni_hoxba Oni_hoxbb Oni_hoxca Oni_hoxda Oni_hoxdb

Figure S1. Number of loci per Mb and coverage of repetitive elements in seven *hox* cluster of (a) *Cynoglossus semilaevis*, (b) *Scophthalmus maximus*, (c) *Sparus aurata* and (d) *Oreochromis niloticus*. The repetitive elements analyzed were Retroelements, DNA transposons, small RNA, rolling circles, satellites, simple repeats and unclassified repetitive sequences (Unknown).

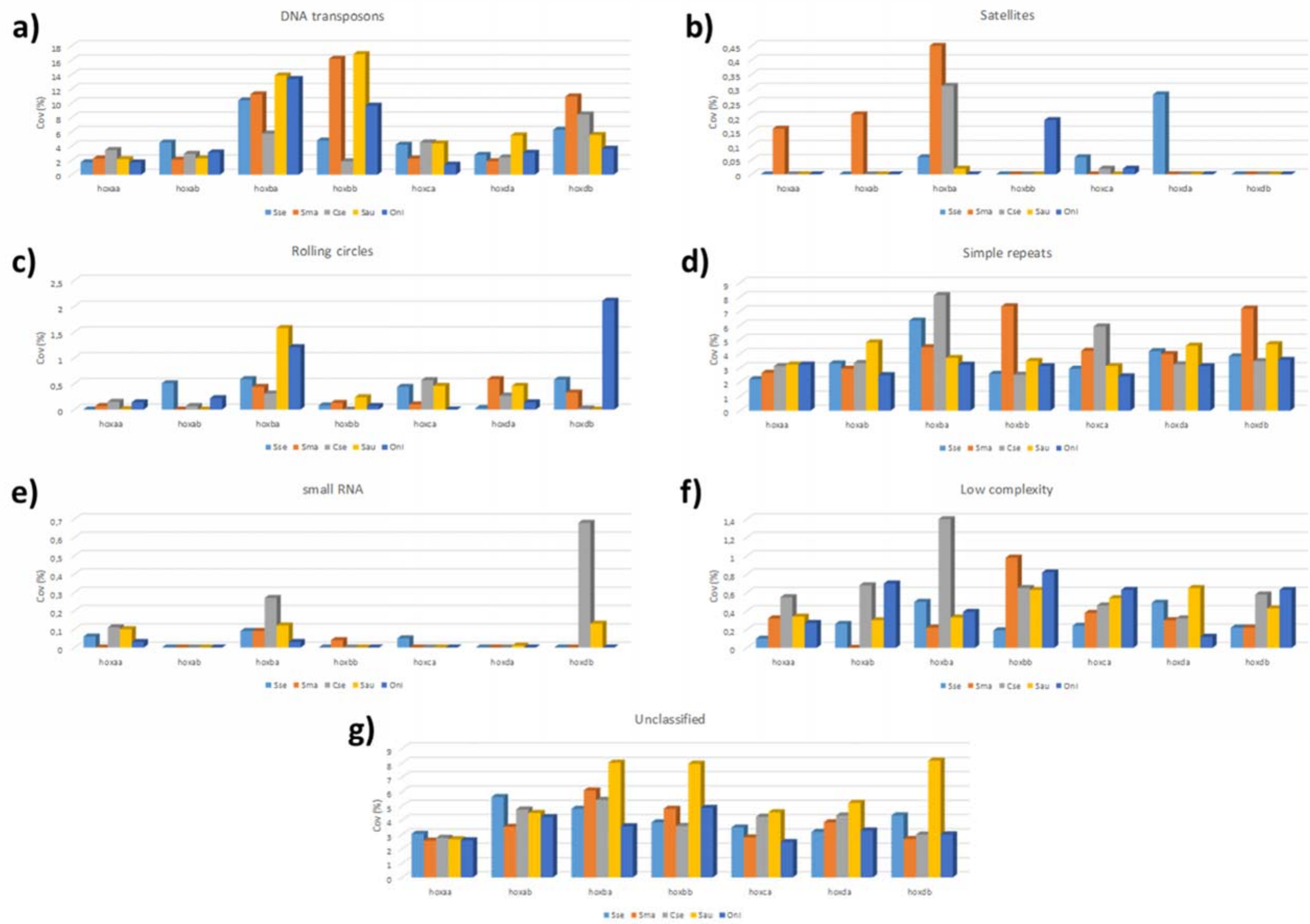

a)
DNA transposons
b)
Satellites
c)
Rolling circles
d)
Simple repeats
e)
small RNA
f)
Low complexity
g)
Unclassified
Cov (%)
hoxaa
hoxab
hoxba
hoxbb
hoxca
hoxda
hoxdb
Sse
Sma
Cse
Sau
Oni

Figure S2. Coverage (%) of (a) DNA transposons, (b) satellites, (c) rolling circles, (d) simple repeats, (e) small RNA, (f) low complexity and (g) unclassified repetitive sequences (Unknown) in seven *hox* cluster of five fish species: *Solea senegalensis*, *Scophthalmus maximus*, *Cynoglossus semilaevis*, *Sparus aurata* and *Oreochromis niloticus*.

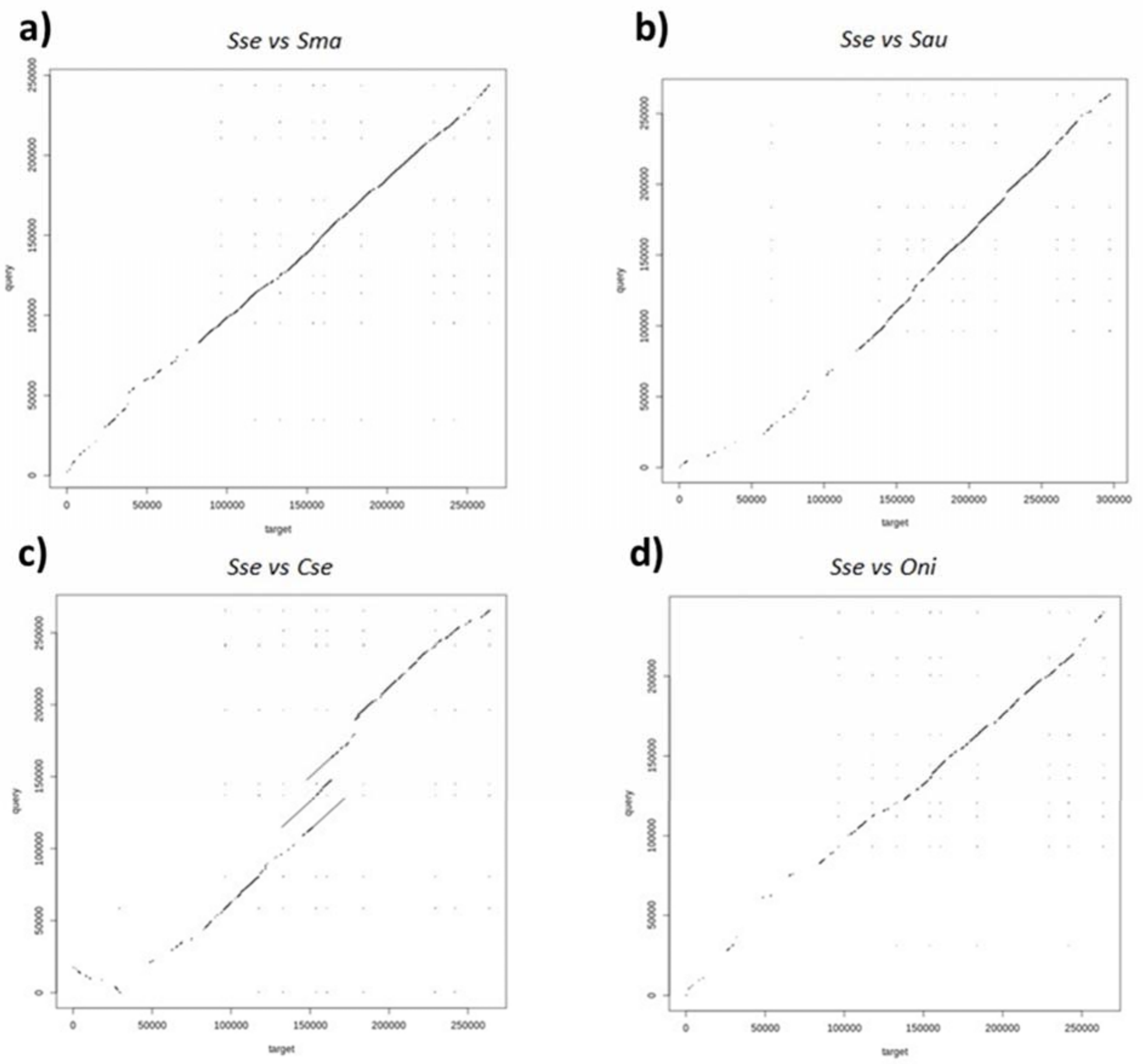

a)
Sse vs Sma
b)
Sse vs Sau
c)
Sse vs Cse
d)
Sse vs Oni
query
target
0
50000
100000
150000
200000
250000
300000

Figure S3. Nucleotide dot plots of the *hoxba* cluster sequence stretch between *Solea senegalensis* and (a) *Scophthalmus maximus*, (b) *Sparus aurata*, (c) *Cynoglossus semilaevis* or (d) *Oreochromis niloticus*.

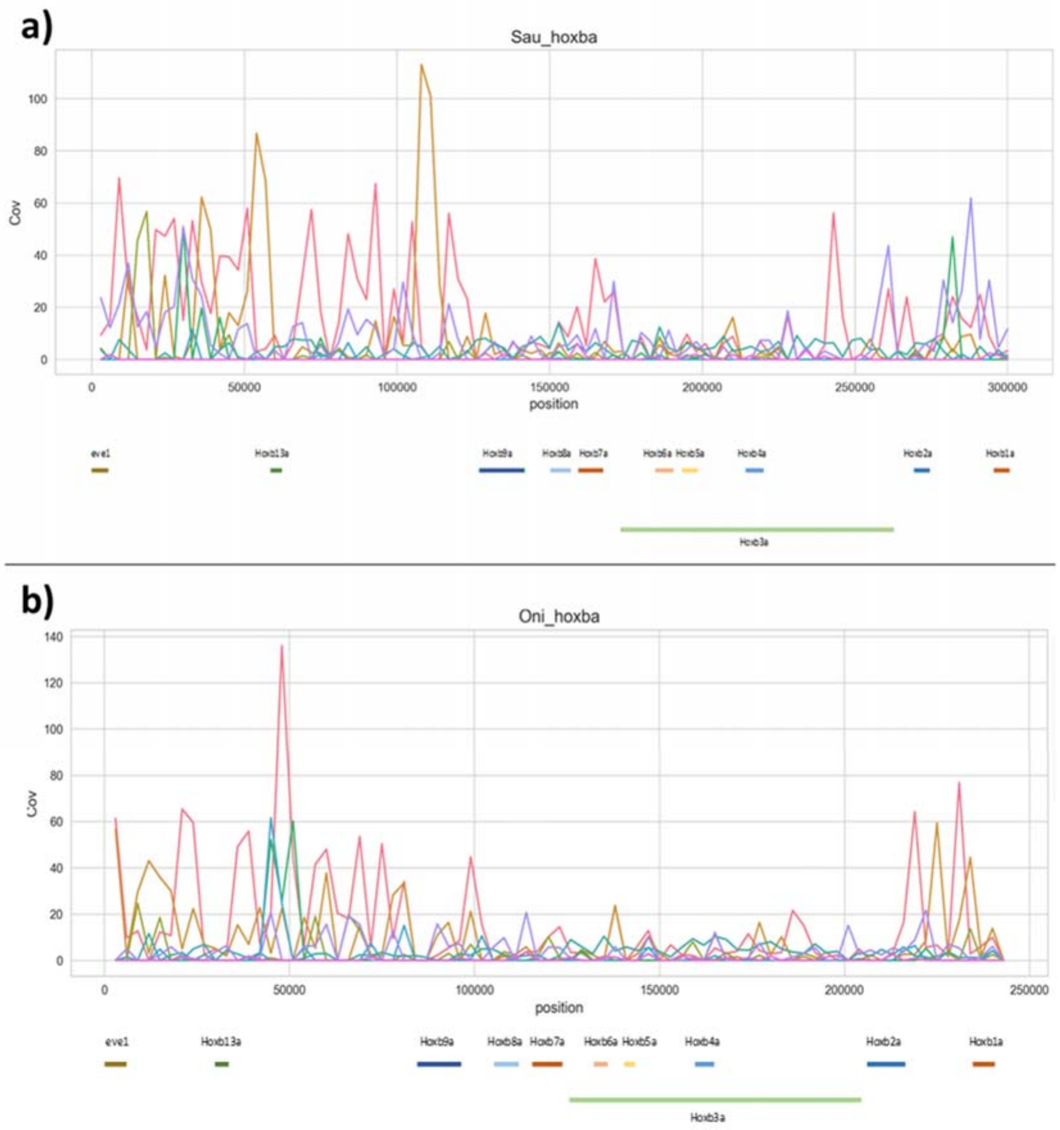

a)
Sau_hoxba
Cov
position
0
20
40
60
80
100
50000
100000
150000
200000
250000
300000
eve1
Hoxb13a
Hoxb9a
Hoxb8a
Hoxb7a
Hoxb6a
Hoxb5a
Hoxb4a
Hoxb2a
Hoxb1a
Hoxb3a
b)
Oni_hoxba
Cov
position
0
20
40
60
80
100
120
140
50000
100000
150000
200000
250000
eve1
Hoxb13a
Hoxb9a
Hoxb8a
Hoxb7a
Hoxb6a
Hoxb5a
Hoxb4a
Hoxb2a
Hoxb1a
Hoxb3a

Figure S4. Abundance of repetitive elements, measured as coverage (%), along the *hoxba* cluster sequence of two teleost species (sliding window 3kb): (a) *Sparus aurata* and (b) *Oreochromis niloticus*. Repetitive elements analyzed were: DNA transposon (light red), LINEs (light orange), LTRs (light green), RC/Helitron (green), Simple repeat (blue-green), SINE (sky-blue), Unknown (purple) and low complexity sequences (pink).

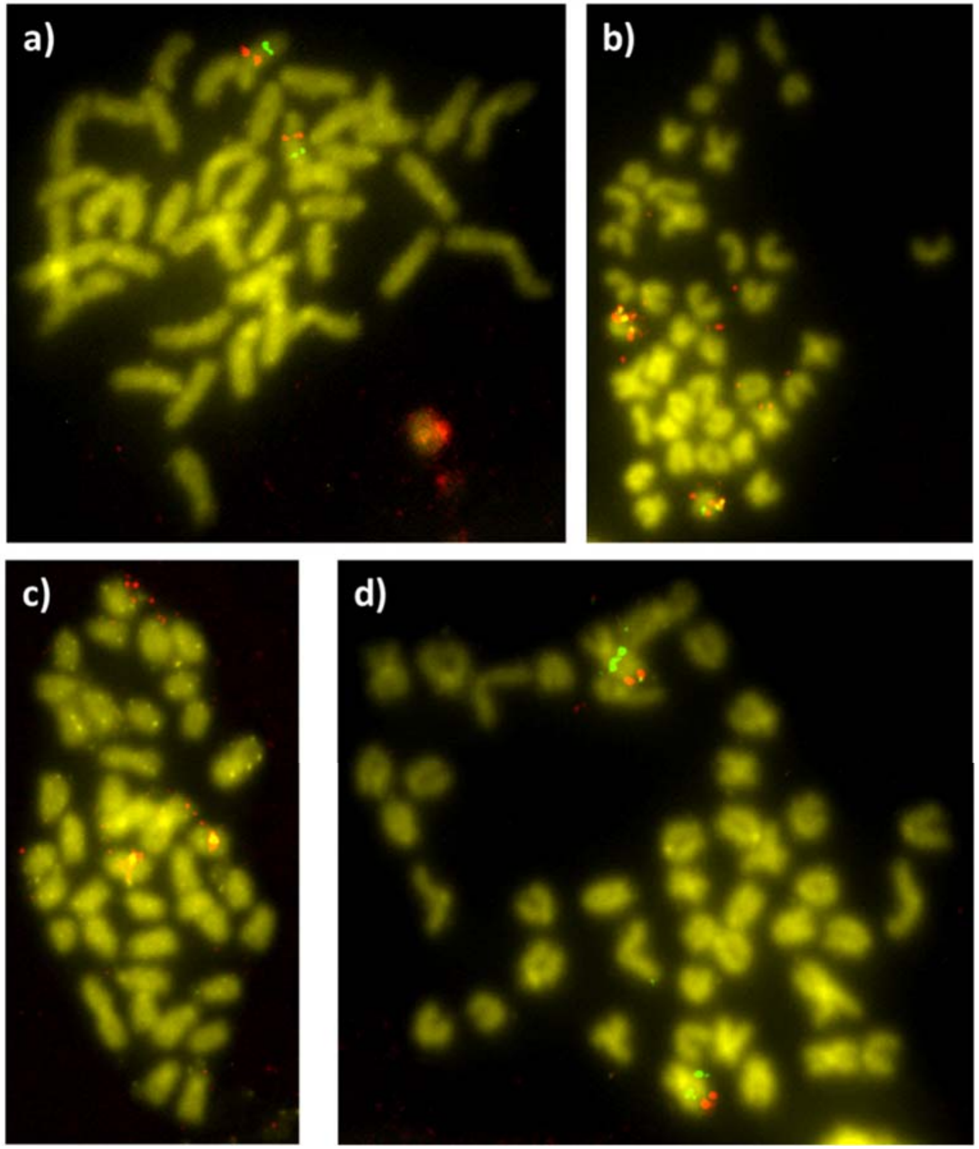
a)
b)
c)
d)

Figure S5. BAC-FISH of among *hox*-bearing BAC clones and BAC probes used as chromosome markers for *Solea senegalensis* karyotype: a) *hoxab* (red)-BAC 54E18 (green); b) *hoxbb* (green)-BAC 38B21 (red); c) *hoxca* (green)-BAC 4M14 (red); d) *hoxda* (green)-BAC 36J2 (red).

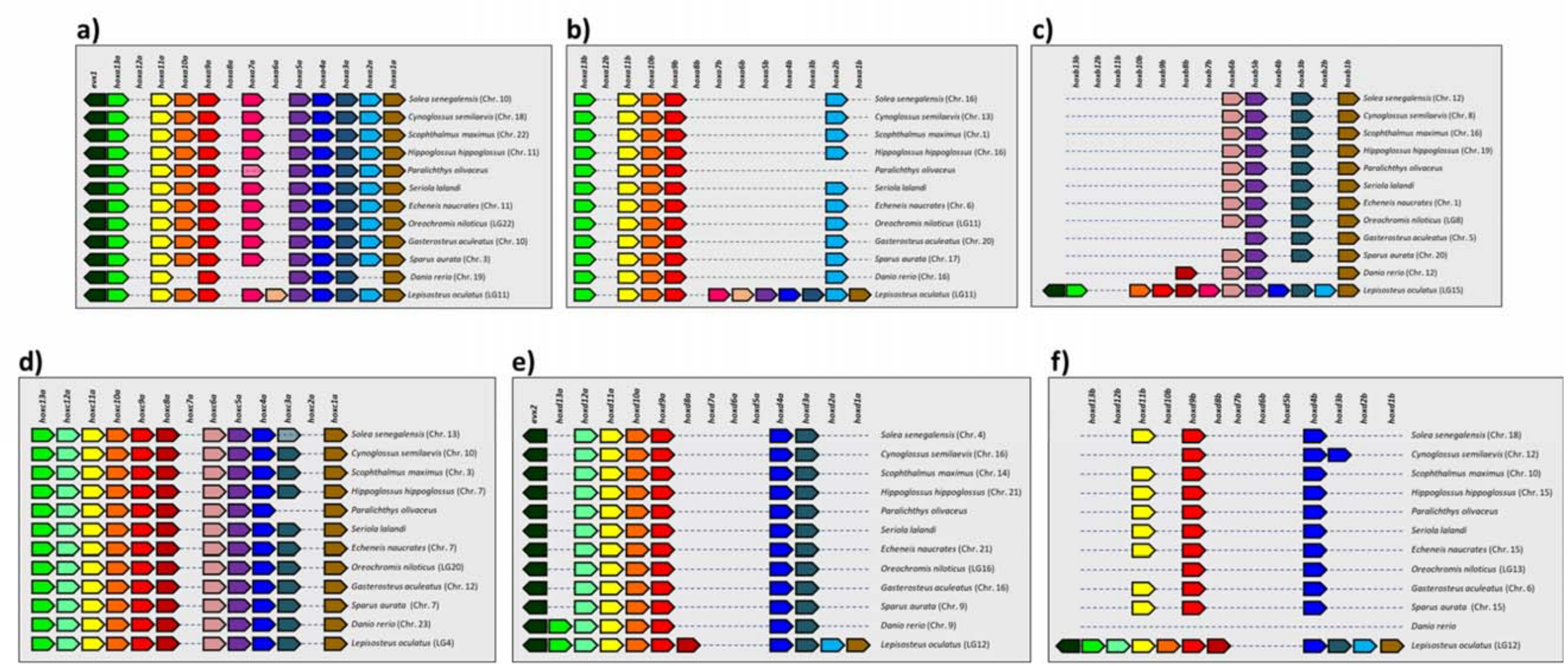

Figure S6. Schematic representation of the (a) *hoxaa*, (b) *hoxab*, (c) *hoxbb*, (d) *hoxca*, (e) *hoxda* and (f) *hoxdb* clusters synteny among different fish species. Same colors represent same genes. Transparent boxes represent genes that were positioned by means of different scaffolds.

Table S2. Polymorphisms detected within teleost species referred to the consensus arrangement of each cluster.

**Cluster**	**Consensus arrangement (5′-3′)***	**Polymorphism**
hoxaa	*evx1-hoxa13a-hoxa11a-hoxa10a-hoxa9a-hoxa7a-hoxa5a-hoxa4a-hoxa3a-hoxa2a-hoxa1a*	Absence of *hoxa10a*, *hoxa7a* and *hoxa2a* in *D. rerio*
hoxab	*hoxa13b-hoxa11b-hoxa10b-hoxa9b-hoxa2b*	Absence of *hoxa2b* in *P. olivaceus*
hoxba	*eve1-hoxb13a-hoxb9a-hoxb8a-hoxb7a-hoxb6a-hoxb5a-hoxb4a-hoxb3a-hoxb2a-hoxb1a*	Inversion of *eve1* and *hoxb13a* in *C. semilaevis*<br>Absence of *hoxb7a* in *C. semilaevis*, *H. hippoglossus* and *P. olivaceus*<br>Absence of *hoxb8a* in *G. aculeatus*<br>*eve1* pseudogenization in *S. aurata*
hoxbb	*hoxb6b-hoxb5b-hoxb3b-hoxb1b*	Absence of *hoxb6b* in *G. aculeatus*<br>Absence of *hoxb3b* in *D. rerio* and presence of *hoxb8b*
hoxca	*hoxc13a-hoxc12a-hoxc11a-hoxc10a-hoxc9a-hoxc8a-hoxc6a-hoxc5a-hoxc4a-hoxc3a-hoxc1a*	Absence of *hoxc3a* in *P. olivaceus*
hoxda	*evx2-hoxd12a-hoxd11a-hoxd10a-hoxd9a-hoxd4a-hoxd3a*	Presence of *hoxd13a* in *D. rerio*
hoxdb	*hoxd11b-hoxd9b-hoxd4b*	Absence of *hoxd11b* in *C. semilaevis* and *O. niloticus*<br>Duplication of *hoxd4b* in *C. semilaevis*<br>Cluster absence in *D. rerio*

* For knowing the specific gene orientation, see figure 5 and supplementary material 6.

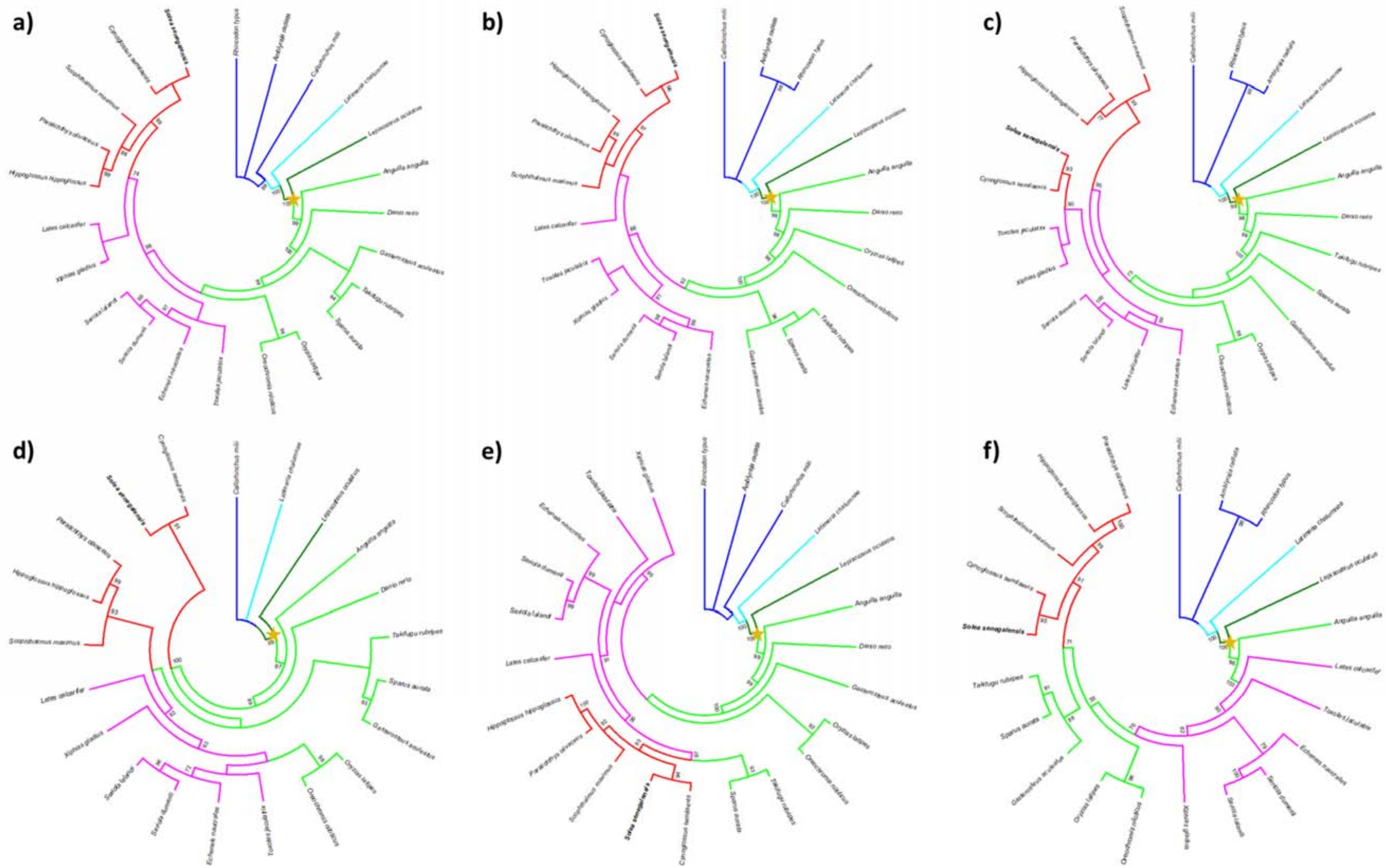
a)
b)
c)
d)
e)
f)

Figure S7. Phylogenetic trees constructed from clusters *hoxaa* (*hoxa1a*, *hoxa3a*, *hoxa4a*, *hoxa5a*, *hoxa9a*, *hoxa11a*, *hoxa13a*, *evx1*), *hoxab* (*hoxa9b*, *hoxa10b*, *hoxa11b*, *hoxa13b*), *hoxbb* (*hoxb1b*, *hoxb5b*), *hoxca* (*hoxc5a*, *hoxc6a*, *hoxc8a*, *hoxc9a*, *hoxc10a*, *hoxc11a*, *hoxc12a*, *hoxc13a*), *hoxda* (*hoxd3a*, *hoxd4a*, *hoxd10a*, *hoxd12a*, *evx2*) and *hoxdb* (*hoxd4b*, *hoxd9b*). Blue lines: Chondrichthyes lineage; cyan lines: Sarcoptherygii lineage; dark green lines: non-Teleostei Actinoptherygii lineage; light green + pink + red lines: Teleostei lineage; pink + red lines: Carangaria lineage; red lines: Pleuronectiformes lineage. Yellow star denotes the teleost-specific genome duplication (TSGD) event.
